# Effect of the Addition of Soluble Dietary Fiber and Green Tea Polyphenols on Acrylamide Formation and In Vitro Starch Digestibility in Baked Starchy Matrices

**DOI:** 10.3390/molecules24203674

**Published:** 2019-10-12

**Authors:** José David Torres, Verónica Dueik, David Carré, Pedro Bouchon

**Affiliations:** 1 Department of Chemical and Bioprocess Engineering, Pontificia Universidad Católica de Chile, P.O. Box 306, Santiago 6904411, Chile; jdtorres2@uc.cl (J.D.T.); vpdueik@uc.cl (V.D.); 2 Comercial e Industrial SOLUTEC Ltda. Almirante Churruca 3130, Santiago 8370653, Chile; dcarre@solutec-chile.cl

**Keywords:** starch digestibility, acrylamide, soluble dietary fiber, green tea, polyphenols, baking

## Abstract

Starch digestibility may be affected by food microstructural changes, as well as by specific interactions with some biomolecules, such as soluble dietary fibers (SDFs). It is well-known that acrylamide (AA) is a toxic and potentially carcinogenic compound formed in starchy food products processed at temperatures above 120 °C. This study aimed to investigate the effect of the addition of SDF and green tea polyphenols (GTP) on AA formation and in vitro starch digestibility in baked starchy matrices. The formulations were prepared using gluten and wheat starch, ensuring ~40 ± 2% (wet basis, w.b.) moisture in the doughs. In some samples, 7.5% (dry basis, d.b.) of starch was replaced with inulin (IN), polydextrose (PD) or partially hydrolyzed guar gum (PHGG), and/or with GTP at 1% (d.b). Acrylamide was determined by gas chromatography–mass spectrometry, and the in vitro starch digestibility using the Englyst method. The GTP was able to reduce AA content by ~48%, and a combination of IN-GTP allowed it to be reduced by up to ~64%, revealing the lowest rapidly available glucose content (~17 mg/g glucose). While a PD-GTP mixture reduced the AA content by around ~57% and gave the highest unavailable glucose fraction (~74 mg/g glucose) compared to the control. This study showed how functional ingredients could be used to develop successfully healthier starchy bakery foods.

## 1. Introduction

Starch is the most important carbohydrate in the human diet and provides a relevant source of energy [1]. However, overconsumption of starchy foods is of concern, as this is associated with type 2 diabetes, which is one of the world’s most prevalent and morbid chronic diseases [2,3]. In the food industry, starch is used as a thickening, a gelling, or a structure-forming agent. Moreover, it is used in formulated products, which are then fried or baked [4,5]. Most of the desired properties of starch are triggered when starch granules are heated in excess water. Under these circumstances, the crystalline structure of the granule is disrupted, allowing amorphous regions to become more accessible to water and swell, increasing their susceptibility to enzymatic degradation [4,6].

Starch digestibility may be affected by the microstructural changes of foods, the amylose-amylopectin ratio, as well as the interaction with some biomolecules, among others [6]. Accordingly, it has been proposed that diabetes could be controlled by modifying the dietary intake of carbohydrates, following these approaches [7]. To measure starch digestibility, Englyst et al. [8] developed an in vitro enzymatic procedure, which allows the rapidly available glucose (RAG), the slowly available glucose (SAG) and the unavailable glucose (UG) fractions to be identified. Diets with a high amount of RAG are related to type 2 diabetes, while the consumption of foods with SAG, although not always resulting in a low-glycemic response, might have several beneficial effects [9,10].

As previously mentioned, one strategy to reduce starch digestibility in starchy foods is the interaction with other components, such as soluble dietary fibers (SDFs), which are non-starch polysaccharides or hydrocolloids that are resistant to human enzymatic digestion and which may promote bowel health by preventing constipation and diverticular disease [11]. It has been proposed that SDF could reduce starch digestibility by interacting with starch or by limiting water availability in starchy products [12]. According to Brennan et al. [13], and Dikeman et al. [14], some SDFs induce a high viscosity in the chyme, which may change starch digestibility rates. Although there is a consensus that an increase in chyme viscosity caused by SDF could modify starch digestibility, it remains unclear how the different types of SDFs may affect the activity of enzymes during starch digestion [15]. Inulin (IN) is an SDF composed of a mixture of fructose chains that vary in length, and which is found naturally in chicory roots [16]. Polydextrose (PD) is a synthetic SDF composed of glucose, sorbitol, and citric acid; it is highly soluble in water, odorless, and colorless in solution. It has been approved by the U.S. Food and Drug Administration to be used as a bulking agent, as well as a sugar or fat substitute in food products [11]. Guar gum is an SDF that is highly viscous and water-soluble, and which is obtained from seeds of *Cyamopsis tetragonolobus*. In its natural form, it cannot be incorporated at high levels in foods, as it affects the organoleptic properties [17]. Consequently, enzymatic hydrolysis is carried out to produce partially hydrolyzed guar gum (PHGG), which is less viscous. PHGG is used as a thickening and stabilizing agent in many processed food products, including: ketchup, creams, beverages, confectionery and bakery products [18]. 

Baking is a complex thermal process that involves many physical, chemical, and biochemical changes that occur inside starchy foods, affecting their physical and nutritional properties [5]. Among these changes, the most relevant include starch gelatinization, protein denaturation, the liberation of carbon dioxide from leavening agents, volume expansion, water activity (*a_w_*) reduction, non-enzymatic browning, and the formation of heat-induced contaminants, such as furan or acrylamide [19,20]. Acrylamide (AA) is a chemical compound with a low molecular weight formed during thermal processing as an intermediate product of the Maillard reactions, principally through the reaction between the amino acid asparagine and reducing sugars, such as glucose or fructose [21]. AA is considered a genotoxic and potentially carcinogenic compound by the International Agency for Research on Cancer [22]. The highest concern about AA intake comes from cereal-based products such as: biscuits, crackers, or bread, which are baked and widely consumed throughout the world [23].

Various efforts to reduce the AA formation in bakery products have been carried out; the alternatives vary from the addition of enzymes to the application of thermal processes at reduced temperatures [21,24]. More recently, some studies have focused on the modification of formulations mixing SDF and phytochemicals [20,22]. As reported by Zeng et al. [25] and Passos et al. [26], SDFs may reduce the AA formation in starchy products, probably owing to their long molecular chains and numerous functional chemical groups that would interact and inhibit AA reactants, as well as by their capacity to reduce the *a_w_* in starchy formulations, which could decrease non-enzymatic browning reactions. However, these effects are still not fully understood.

The phytochemicals that have been used to reduce AA content include polyphenols obtained from different agro-industrial sources [27]. In more recent times, the use of green tea polyphenols (GTP) derived from the leaf extracts of *Camellia Sinensis*, have aroused the interest of the food industry, due to their positive effects on human health, mainly as an anti-diabetic, anti-obesity agent and their ability to scavenge advanced glycation end-products [27,28,29]. Most of the GTP are flavanols, namely catechins (e.g., (+)-catechin gallate, (+)-gallocatechin, (+)-gallocatechin gallate, (−)-epicatechin, (−)-epicatechin gallate, (−)-epigallocatechin, and (−)-epigallocatechin gallate). GTP presents the highest antioxidant capacity among other types of polyphenols [30,31]. Some researchers have recently focused their studies on the role of GTP in the digestive properties of starch, as well as on their role in non-enzymatic browning reactions, which promote AA formation during baking [32,33]. As reported by Goh et al. [34], catechins would interact mainly with the amorphous regions of starch, which are usually more readily hydrolyzed by amylolytic enzymes, which could reduce starch digestibility. Furthermore, due to their chemical nature, catechins may interact with amino acids and reducing sugars, inhibiting AA formation in starchy foods [35,36]. However, it is still necessary to understand and clarify these outcomes.

Therefore, the aim of this study was to investigate the effect of the addition of SDF (IN, PD, and PHGG) and GTP on AA formation and in vitro starch digestibility in baked starchy matrices.

## 2. Results

### 2.1. Physicochemical Behavior of the Soluble Dietary Fibers (SDFs) Used in Starchy Matrices 

Table 1 shows the water-holding capacity (WHC) and apparent viscosity characteristics of the different SDFs used in this study. WHC and viscosity are essentials properties of SDFs, which have a significant influence on numerous industrial food applications [37,38]. According to Mancebo et al. [39], WHC provides an estimate of the amount of water that can be captured by SDF in starchy foods. The said amount of water would depend on the SDF structure as well as its chemical and physical nature. In this study, the highest WHCs were obtained to IN and PHGG. They were ~35 and ~27% higher than starch, which showed a WHC of 1.85 g water/g dry solids. WHC in this study agrees with Collar et al. [40] who reported a value of ~2.06 (g water/g dry solids) for IN used in bread doughs. Meanwhile, Rosell et al. [38] found significantly higher values of WHC for IN ~ 11.05 ± 0.49 (g water/g dry solids) when assessing the hydration properties of several SDFs with potential applications in bakery products. As reported by Mancebo et al. [39] WHC values were 7.96 and 7.26 (g water/g dry solids) for IN and PD, respectively, and added them into sugar-snap cookies. 

With respect to the apparent viscosity of SDF, the highest value (at 30 °C) was obtained in PHGG (~8 mPa·s), followed by PD (~5 mPa·s) and by IN (~2 mPa·s). The viscosity of the SDFs in this study coincides with Rosell et al. [38] who found values of ~2 mPa·s for IN. These results are also similar to those reported by Kaur and Das [41] who studied the viscosity of suspensions with SDF. 

### 2.2. Changes of Water Activity (a_w_) and Moisture Content of Starchy Matrices 

**Figure 1:**
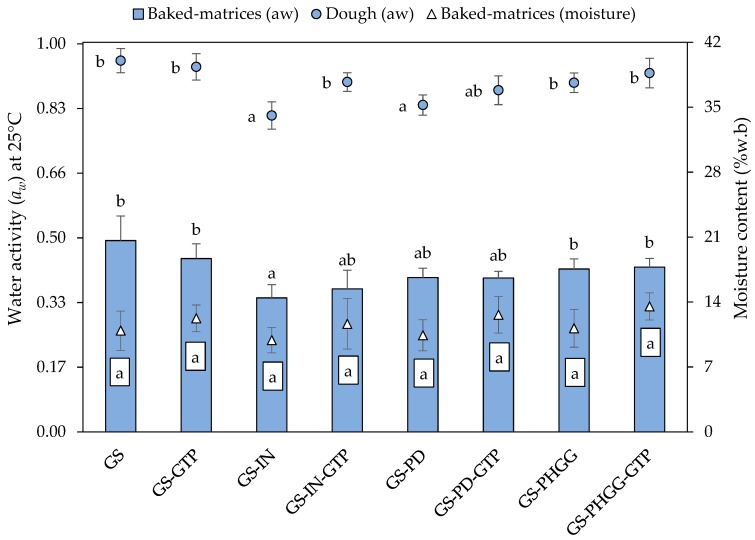
Water activity (*a_w_*) at 25°C of doughs and baked matrices, and moisture content (%w. b) of baked matrices. Different superscripts denote significant differences (*p* < 0.05). Data are means ± standard deviation (*n* = 6). GS: gluten-starch; IN: inulin; PD: polydextrose; PHGG: partially hydrolyzed guar gum; GTP: green tea polyphenols.

Figure 1 shows the effects of SDF and GTP addition on *a_w_* at 25 °C in the different doughs and baked matrices. The *a_w_* value of the control dough (model matrix made of gluten and starch, referred as G-S) was 0.953 ± 0.031, and decreased significantly up to 0.812 ± 0.035 and 0.839 ± 0.026 in doughs that only contained either IN or PD only, respectively. The sample with PHGG reduced its mean value up to 0.896 ± 0.025, but the difference was not significant (*p* > 0.05). These results are consistent with a possible protective effect of SDF in holding water within the dough structure, following the WHC results (Table 1). Similar results were reported by Rodríguez-García et al. [42], who found that *a_w_* decreased significantly in short dough biscuits (*p* < 0.05) when SDF was incorporated.

Most starchy foods are usually baked up to a final moisture ≤15%. In our set of experiments, the different samples were dehydrated up to similar levels, below this limit, as shown in Figure 1. As can be seen, all baked samples had an *a_w_* < 0.6. Furthermore, baked samples containing SDFs had a significantly lower *a_w_*, compared to the control, an aspect that could be linked to a higher WHC. According to Labuza et al. [43], *a_w_* is a measure of the amount of water available for chemical reactions, as well as microbial growth in starchy foods; therefore, its measurement is essential in the bakery industry as it is linked with the stability and safety of foods throughout its shelf-life [44]. 

### 2.3. Textural Characteristics of Doughs before Baking

**Figure 2:**
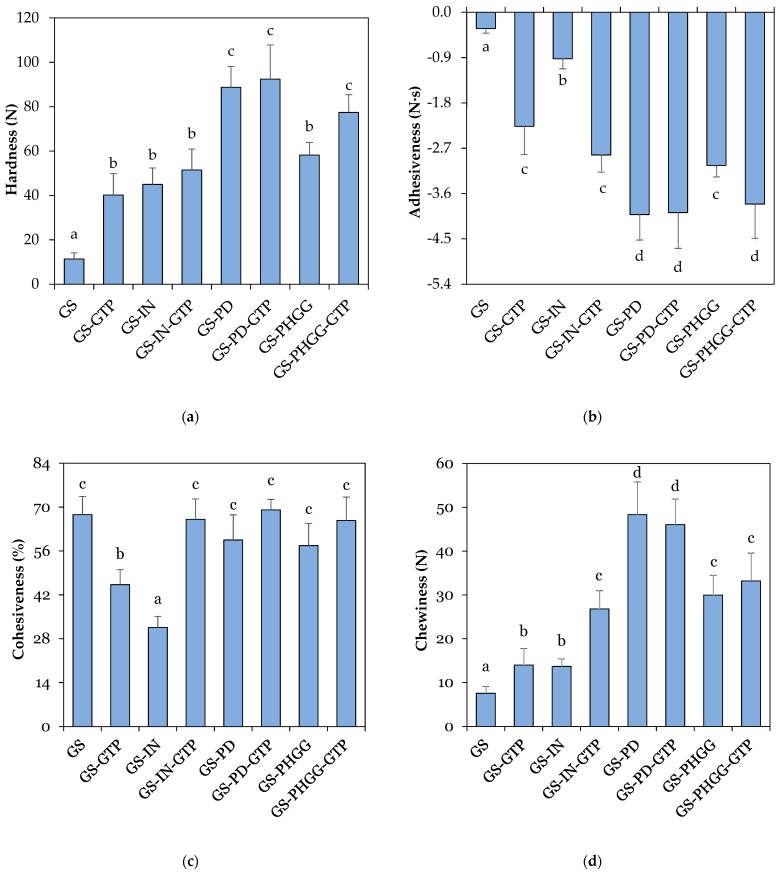
Texture profile analysis of the different doughs used in this study. (**a**) Hardness; (**b**) adhesiveness; (**c**) cohesiveness; (**d**) chewiness. Data are means ± standard deviation (*n* = 6). Different superscripts denote significant differences (*p* < 0.05). GS: gluten-starch; IN: inulin; PD: polydextrose; PHGG: partially hydrolyzed guar gum; GTP: green tea polyphenols.

Textural parameters were assessed instrumentally by means of a texture profile analysis (TPA) [45]. As shown in Figure 2, the hardness of doughs with SDF and GTP varied from ~40 to ~92 N (Figure 2a), while the chewiness ranged from ~13 to ~48 N (Figure 2d), and their adhesiveness fluctuated between −0.9 to −3.9 N·s (Figure 2b). These values differed from the control (*p* < 0.05), the hardness of which was around ~11 N, its chewiness ~7 N, and its adhesiveness close to −0.3 N·s. In this research, the combination of SDF-GTP increased the hardness, chewiness, and adhesiveness in all starchy doughs (*p* < 0.05), obtaining the highest values in formulations with PD and PD-GTP. Doughs with PHGG were more adhesive than those made with IN (*p* < 0.05). Cohesiveness is an essential characteristic of starchy doughs used in bakery products, as it reflects the internal forces that hold the structure together as a whole [46,47]. The cohesiveness of starchy doughs with SDF and GTP ranged from ~31 to ~69% (Figure 2c), while the control showed a value of ~67%. In contrast, cohesiveness decreased in samples that only contained either IN or GTP (*p* < 0.05), respectively. There were no differences in dough cohesiveness when IN, PD, and PHGG were incorporated along with GTP (*p* > 0.05). Overall, all formulations could be adequately laminated and processed. 

### 2.4. Textural Changes of Starchy Matrices after Baking

**Figure 3:**
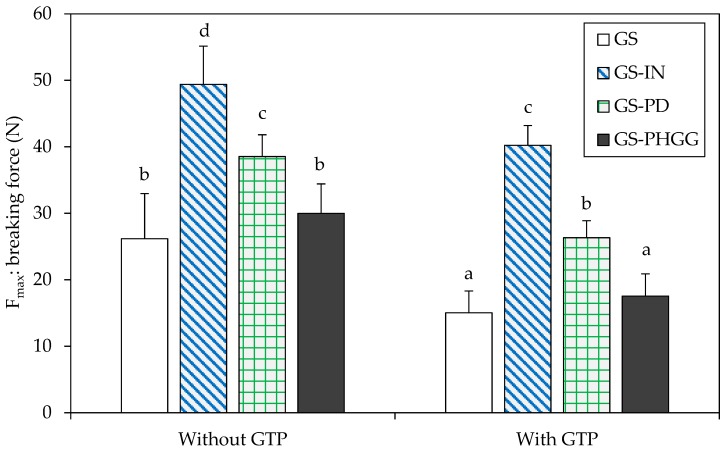
Effect of soluble dietary fibers (SDF) and GTP addition on the hardness (N) of baked matrices. Data are means ± standard deviation (*n* = 6). Different superscripts denote significant differences (*p* < 0.05). GS: gluten-starch; IN: inulin; PD: polydextrose; PHGG: partially hydrolyzed guar gum; GTP: green tea polyphenols.

Hardness, which corresponds to the maximum breaking force produced upon compression of the sample, was used as a textural descriptor [48]. As shown in Figure 3, the addition of IN and PD caused an increase in hardness of around ~70% and ~30%, respectively, with respect to the control (*p* < 0.05). There was no significant increase in hardness in starchy matrices with PHGG (*p* > 0.05). The force required to break the baked matrices was significantly reduced when GTP was added (*p* < 0.05). For instance, in starchy models with PHGG-GTP, PD-GTP, and IN-GTP these reductions were close to ~30%, ~18%, and ~16%, respectively, compared to their counterparts without GTP.

### 2.5. Acrylamide Content of Baked Starchy Matrices

**Figure 4:**
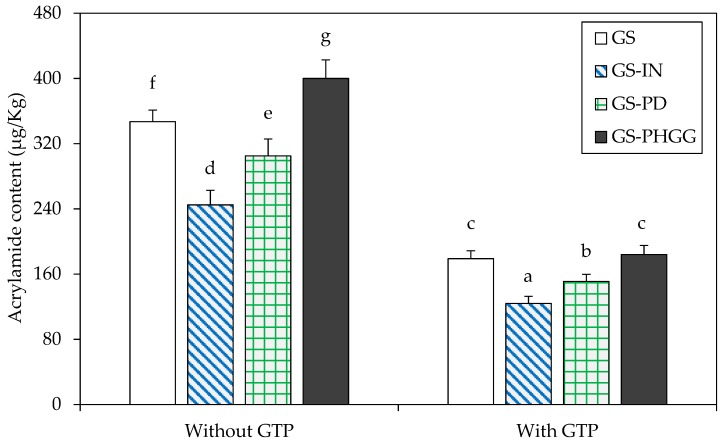
Effect of SDF and GTP addition on acrylamide content (μg/kg dry solids) of baked starchy matrices developed in the study. Data are means ± standard deviation (*n* = 3). Different superscripts denote significant differences (*p* < 0.05). GS: gluten-starch; IN: inulin; PD: polydextrose; PHGG: partially hydrolyzed guar gum; GTP: green tea polyphenols.

Figure 4 shows the effect of SDF and GTP addition on the AA content in baked starchy matrices. The addition of IN and PD reduced AA content by ~29 and ~12%, respectively, compared to the control dough (*p* < 0.05). There was no evidence of AA reduction when PHGG was incorporated individually into the dough (*p* > 0.05). However, a significant decrease in the amount of AA was attained when using PHGG along with GTP (up to ~47% compared to the dough without GTP). In fact, a remarkable decrease in AA content was consistently obtained in all doughs containing GTP. The lowest AA content was obtained in the dough with IN-GTP (~124 μg/kg dry solids), followed by formulations with PD-GTP (~151 μg/kg dry solids) and by those with PHGG-GTP whose AA content was around ~184 μg/kg dry solids (*p* < 0.05), similar to that obtained in the control dough with GTP ~179 μg/kg dry solids.

### 2.6. In Vitro Starch Digestibility of Baked Starchy Matrices

**Figure 5:**
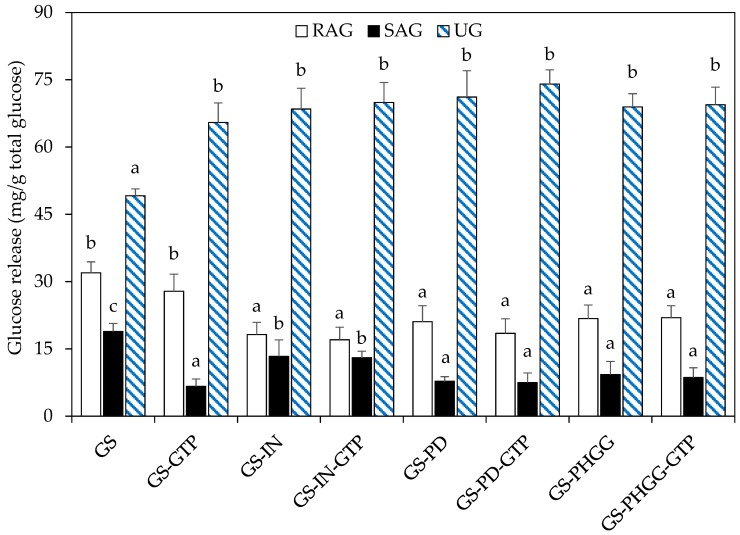
Effect of SDF and GTP addition on rapidly available glucose (RAG, mg/g), slowly available glucose (SAG, mg/g), and unavailable glucose (UG, mg/g) fractions in baked matrices. Data are means ± standard deviation (*n* = 3). Different superscripts denote significant differences in each glucose fraction (*p* < 0.05). GS: gluten-starch; IN: inulin; PD: polydextrose; PHGG: partially hydrolyzed guar gum; GTP: green tea polyphenols.

Figure 5 shows the effect of SDF and GTP addition on in vitro starch digestibility (i.e., the release of glucose fractions) in baked starchy matrices. The addition of SDF led to the reduction of the RAG concentration along with an increase in UG concentration in all samples, compared to the control dough, with or without GTP inclusion (*p* < 0.05). Samples that contained only GTP, did show a significant increase in UG concentration compared to the control matrix (*p* < 0.05). However, even though RAG concentration was lower than the control, the difference was not statistically significant (*p* > 0.05). Considering SAG content, the amount released from starchy matrices with SDF was close to ~8 mg/g total glucose (*p* < 0.05), which was lower than the results obtained in formulations with GTP (~10 mg/g total glucose).

The combined effect of functional ingredients indicated that the lowest value of RAG (~17 mg/g total glucose) was obtained in formulations containing IN-GTP, which in turn raised the UG fraction up to ~70 mg/g total glucose. Similarly, baked starchy matrices with PD-GTP were found to have a RAG content of ~18 mg/g total glucose, along with the highest increase in UG (up to ~74 mg/g total glucose). Likewise, baked starchy matrices with PHGG-GTP presented amounts of RAG and UG fractions of around ~21 and ~69 mg/g total glucose, respectively. Overall, a similar effect on starch digestibility reduction was achieved by mixing, indistinctly, any of the three SDFs along with GTP. 

## 3. Discussion

### 3.1. Textural Characteristics of Starchy Matrices before and after Baking

Texture parameters of dough are decisive for understanding the changes that take place during baking [49]. As previously indicated, an increase in the hardness of dough was observed in samples with SDF, compared to the baked dough control. This phenomenon could be due to a higher development of the protein network promoted by the addition of SDF [50]. As reported by Li et al. [51], wheat dough mixed with SDF exhibited an increase in hardness by up to ~117% compared to the control dough. Meanwhile, Mudgil et al. [18] reported that addition of PHGG in the flour used to make noodles, improved texture parameters such as: hardness, adhesiveness, cohesiveness, and chewiness. These results coincide with outcomes from Sivam et al. [7] who explained that the addition of bioactive ingredients such as SDF and/or GTP in bakery food products may promote appropriate cross-links among wheat proteins via hydrogen bonding not only during dough preparation but also during the baking process, which leads to strengthening of the food structure. 

According to Peressini and Sensidoni [16], Li et al. [45], and Wang et al. [46], the addition of SDF increases wheat-dough consistency, hardness, and stability, by changing its structure as well as water availability, which depends on the physicochemical properties of the SDF. The SDFs used in this research significantly influenced the water balance and hardness of starchy dough (PD > PHGG > IN). The redistribution of moisture inside the dough structure could allow SDF or GTP to interact directly with gluten molecules, generating a more complex network containing gluten, along with functional ingredients that could partially reduce dough extensibility and may be linked to a higher degree of chewiness of the dough [7]. However, it is essential to control the dough hardness, given that an excessive increase in this textural parameter could hinder the sheeting process, thereby lowering baking performance [51].

During baking, starch gelatinization occurs. This is a crucial process that demands both the presence of liquid water and enough heating to rise the temperature above ~55 °C, contingent to the food matrix and the botanical source of starch [6,19]. These transformations, together with the protein network, allow for the setting up of the matrix structure during baking. In addition, water loss leads to the formation of a dehydrated crust, which transits towards the interior, allowing the temperature inside the matrix to rise substantially, thus strengthening the structure [5]. The increase in hardness of baked matrices with SDF could be due to the development of a stronger dehydrated crust, which may change depending on the chemical composition of each ingredient added to the formulation [48,52]. According to Brennan et al. [13], SDF could act as a coating on the microstructure of starchy foods. This fact may explain the increment in the hardness of starchy formulations with IN or PD. On the other hand, the lower degree of hardness in baked matrices with GTP may be determined by the chemical interaction between starch polymers and catechins, through hydrogen bonds that might be reducing the contact with the molecules of gluten, and SDF, weakening the surfaces and inner structure of the starchy matrices [53].

### 3.2. Effect of SDF and Green Tea Polyphenols (GTP) on Acrylamide (AA) Reduction in Baked Starchy Matrices

The decrease of AA content caused by IN and PD could be attributed to their interaction with critical components. These types of SDF have long molecular chains with an abundance of chemical functional groups, which could increase the probability of interaction with amino acids or reducing sugars, decreasing non-enzymatic browning reactions and, therefore, reducing the AA formation during baking [22,26]. It is important to remember that changes of *a_w_* have been linked to AA formation during thermal processing, as the Maillard reactions in starchy foods decreases below *a_w_* < 0.6 and reaches an optimum value at aw~0.66 [43,44].

Results suggest that IN and PD might be promising inhibitors of AA formation during the baking of starchy food products. Nevertheless, there is still a need to understand the possible mechanisms involved in such reduction. Furthermore, PHGG has short chains molecules and did not cause the same effect of AA reduction (*p* > 0.05). Concerning the effects of SDF on AA formation, Zeng et al. [25] evaluated the influence of eight SDF on AA content in starchy model systems. They found reductions between ~30 and ~50% compared to the control. Similarly, Sansano et al. [54] demonstrated the protective effect of SDF on AA formation in starchy matrices processed at 170 °C. They indicated ~59% in AA reduction in formulations with ~0.3% SDF and a decline up to ~85% when SDF was increased by up to ~1%.

As indicated earlier, the AA content was reduced by up to ~48% (*p* < 0.05) in starchy matrices when GTP was incorporated. It has been indicated that baking temperatures cause the degradation of GTP, forming substances of low molecular weight, which due to their antioxidant effects, would interact with free amino acids, reducing non-enzymatic browning reactions [35,55,56,57]. Furthermore, Liu et al. [33] explained that GTP could inhibit AA formation through the entrapment of carbonyl compounds and intervention in the Amadori rearrangement. Similarly, Constantinou and Koutsidis [58], Sansano et al. [59], Kahkeshani [60], Jin et al. [61] and Jiang et al. [62] argued that phenolic compounds may react with sugar fragments and reactive carbonyl compounds, forming adducts through electrophilic aromatic substitution reactions and thus inhibit AA formation. This trend coincides with Fu et al. [36] who studied the effects of epigallocatechin gallate (EGCG) extracted from GTP on AA formation in white bread. They reported that EGCG significantly reduced the AA formation by ~37% compared to the control, due to its antioxidant capacity. Despite this fact, the exact mechanisms about how GTP reduces AA formation are yet to be fully understood. 

Overall, AA values of SDF-GTP formulations were slightly higher than the minimum levels reported in bakery food products by the European Food Safety Authority [63], ranging between 30 and 100 μg/kg of total food, and were significantly lower than those reported by Zhu et al. [64] who found values around ~370 µg/kg in cookies. They obtained results showing that phytochemicals such as aqueous extracts of clove at 4% caused the most significant AA reduction (~50.9%) in cookies, whereas the addition of 2% proanthocyanidins from grape seeds led to the most considerable AA decrease (~62.2%) in starchy models. According to this trend, Pedreschi et al. [20] reported that the addition of 750 mg/kg of tara pod extract to bread reduced AA content almost by ~50%. Moreover, when tara pod extract was added at 1500 mg/kg, AA reduction was close to ~97%. Likewise, Li et al. [65] after adding ~200 mg/kg bamboo leaves antioxidant extract (~25% flavonoids), and ~100 mg/kg GTP (~98% catechins) to the mix for cookies, reported that the AA content decreased by up to 63% and 71%, respectively. Among the mechanisms by which these phytochemicals are supposed to mitigate AA content in starchy foods, free radical scavenging activity is noteworthy, particularly in the utilization of GTP or plant-derived antioxidants [22,31].

### 3.3. Effect of SDF and GTP on In Vitro Starch Digestibility in Baked Starchy Matrices

The addition of SDF led to a reduction in RAG and the SAG concentration in all samples, compared to the control dough, with or without the inclusion of GTP (*p* < 0.05). This fact was reflected by a significant increase in the UG fraction (*p* < 0.05). As reported by Brennan et al. [13], SDF may reduce starch bioaccessibility by changing the microstructure or by limiting water availability in starchy foods. This phenomenon may restrict starch gelatinization and therefore, could decrease starch digestibility. Dhital et al. [1], and Dikeman et al. [14] argued that SDF could build an active gel matrix surrounding starch granules, which would act as a physical barrier, blocking the access of amylolytic enzymes. This trend agrees with Fabek and Goff [15], who additionally suggested that SDF can increase viscosity during starch digestion, restricting the amylolysis. In earlier research, Brennan et al. [12] assessed the effect of SDF on starch digestibility in wheat bread and analyzed the microstructural changes. They indicated that those types of SDF might delay the enzymatic hydrolysis of starch by modifying its microstructure while increasing the chyme viscosity. In line with these findings, Sasaki et al. [66] reported the existence of complexities between SDF-amylopectin, and suggested that this interaction may cause the inhibition of enzymatic hydrolysis. Meanwhile, Schuchardt et al. [10] indicated that the presence of IN in cookies limited starch gelatinization and increased the resistance to enzymatic attack, lowering the levels of starch digestibility. Nevertheless, more studies are needed to obtain a better understanding of the interactions between SDF or other components and starch that affect the digestibility of the latter [67].

When GTP has been added alone, a significant reduction of the SAG fraction, compared to the control, was obtained, which was translated into a considerable increase of the UG fraction. However, no significant differences regarding the RAG fraction were noted. As reported by Liu et al. [29], and Yilmazer-Musa et al. [32] GTP may inhibit amylolytic enzymes, this could diminish starch digestibility, thus increasing the UG fraction. Likewise, Xiao et al. [68] explained that the hydrophilic groups of GTP might promote interactions with side chains of amylopectin, consequently reducing starch bioaccessibility. Accordingly, Dueik and Bouchon [69] reported that olive leaf polyphenol extracts added to starch matrices affected starch gelatinization, which in turn could affect its digestibility. However, the underlying mechanisms responsible for the reduction of starch digestibility, caused by GTP or antioxidants, are yet to be clearly defined.

## 4. Materials and Methods 

### 4.1. Materials and Supplies 

Matrices were prepared using vital wheat gluten (5.91% moisture) and native wheat starch (12.33% moisture), which were obtained from Roquette^®^ France (Lestrem). Inulin (Fibruline^®^ I, ≥90%; 4.32% moisture) was acquired from Cosucra Groupe Warcoing S.A., Pecq, Belgium. Polydextrose (Fiber^®^ C, ≥ 95%; 1.46% moisture), was purchased from Baolingbao Biology, Co., Ltd., Yucheng, Shandong, China. Partially hydrolyzed guar gum (Sunfiber^®^, ≥85%; 7.31% moisture), was obtained from Taiyo International Inc., Minneapolis, MN, USA. Green tea polyphenols (2.95% moisture, Sunphenon 90M^®^), containing 88.81% (d.b) polyphenols (of which catechins were ~75.72%, with a percentage of epigallocatechin gallate ~45.77% attained by high-performance liquid chromatography (HPLC), according to the producer´s statement), were purchased from Taiyo International, Inc., Minneapolis, MN, USA. Distilled water was obtained from Sumilab S.A., Santiago, Chile.

Pepsin-P7000, amyloglucosidase-A7095, pancreatin-7545, along with guar gum No-G4129, reactive benzoic acid No. 242381, acetic acid 1M, No. 537020 and hydrochloric acid No. 433160 (Sigma-Aldrich, St. Louis, MO, USA), invertase-390203D (VWR International Ltd., Poole, UK), potassium hydroxide in lentils No. PO-1300, sodium acetate 3-Hydrate No. SO-1400 (Winkler Ltda. Santiago, Chile), were used for in vitro digestibility assays. 

AA (2-propene amide, >99.5%, Sigma-Aldrich, St. Louis, MO, USA) labeled d3-acrylamide (>98%, Polymer Source Inc., Dorval, QC, Canada), and acetonitrile (HPLC grade, Rathburn Chemicals Ltd., Walkerburn, Scotland), were used to quantify AA.

### 4.2. Assessment of Physicochemical Properties of SDF

#### 4.2.1. Water-Holding Capacity (WHC)

The WHC is defined as the weight of water that is retained by 1 g of dry material under specified conditions of temperature, time soaked, and speed of centrifugation [40]. The WHC of SDF was measured using the centrifugation method described by Mancebo et al. [39] with some modifications. Samples (~3 g) were dispersed in 25 mL of distilled water and placed in pre-weighed centrifuge tubes. The dispersions were stirred and left at 25 °C for 24 h, in conditions of excess water keeping a sample–water ratio of 1:12. After that, they were centrifuged at 3000 rpm (704× *g*) for 15 min, using a TDL-50B centrifuge (Ningbo Hinotek Technology Co., Ningbo, China). The supernatant was discarded, and WHC was calculated according to Equation (1):

(1) $$\mathrm{WHC}\;(\frac{g\;\mathrm{water}}{g\;\mathrm{solids}\;\mathrm{of}\;\mathrm{sample}})=\frac{\left(P_{w}-P_{o}\right)-\left(P_{s}-P_{o}\right)}{P_{m}}$$

where: P_o_ is the weight of the empty tubes, P_m_ is the weight of the sample, P_w_ is the weight of the tubes with the wet sample, and P_s_ is the weight of the tubes with the dry sample.

#### 4.2.2. Apparent Viscosity

The Mitschka method with some modifications was used to measure apparent viscosity of the SDF [70]. Briefly, solutions at 5% were prepared [41]. Solutions were maintained at 30 °C with constant stirring for 5 min. Next, the solutions were analyzed using a Brookfield digital viscometer (model LVDV-E 115, Middleborough, MA, USA). A water bath (model ULA-40Y, Brookfield Engineering Laboratories, Inc. Stoughton, MA, USA), connected to a viscometer was used to maintain a constant temperature during the analysis. Measurements were carried out at 100 rpm with a shear rate of 30 s^−1^, using spindle 1. Results were expressed in centipoise (mPa·s).

### 4.3. Samples Preparation 

Structured matrices, based on vital gluten, wheat starch, and SDF (IN, PD, and PHGG) were prepared, ensuring a final moisture in the dough between ~38% and 42% (w.b). Another set of experiments was performed, with the same components and adding GTP (catechins). The added water was adjusted considering the water content of the ingredients. The control dry mix contained vital gluten (12% d.b) and native wheat starch (88% d.b). Additional samples were prepared by replacing starch with SDF and/or GTP, which were added up to 7.5 and/or 1.0 g/100 g dry solids, respectively (see Table 2).

The ingredients and their amounts were chosen based on the literature review [16,48,53], and according to preliminary experiments, which ensured an adequate dough structure formation. The gluten level was kept constant since it ensured a sheeted dough with the elasticity and extensibility properties required, without the need to incorporate additional ingredients. SDF concentration was fixed based on results obtained from in vitro starch digestion assays, which revealed that lower amounts of SDF did not have an important effect on starch digestibility, whereas higher levels could affect the dough structure.

Dry ingredients were first mixed for 2 min using a 5K5SS mixer (Kitchen-Aid, St. Joseph, MI, USA) equipped with a K5AB flat beater at 40 rpm. Then, half of the water required was added at 15 °C while mixing for 1 min. After mixing for 3 min, the remaining amount was added at 90 °C while mixing for 2 min. After that, the dough was allowed to rest for one hour inside a plastic film. Then, the dough was sheeted using an LSB516 dough sheeter (Doyon, Saint-Côme-Linière, QC, Canada), until obtaining a final thickness of 2 mm. The sheeted dough was cut into a square-shaped (4 × 4 cm^2^) ensuring a constant weight (2.7 ± 0.2 g). The plates were kept in plastic films to prevent dehydration before baking [48]. Baking was carried out in an electric forced convection oven (model Self Cooking Center^®^, Rational International AG, Landsberg, Germany), using 0% relative humidity at a fixed temperature (170 °C), during 45 min, ensuring a final moisture of ~11 ± 2% (w.b) in all samples [71]. Baked matrices were incorporated in self-closing polypropylene bags and then stored under controlled conditions of temperature and relative humidity for a maximum time of up to 2 h before the physicochemical analysis.

### 4.4. Mechanical and Physical Methods

#### 4.4.1. Moisture Content of Starchy Matrices

The moisture content was analyzed using the standard method of the Association of Official Analytical Chemists [72]. Baked samples were dried up to constant weight at 105 °C for 24 h in a forced air oven (model LDO-080F, Labtech Inc., Namyangu, Korea). 

#### 4.4.2. Water Activity (*a_w_*) of Starchy Matrices

A portable instrument Novasina™ ms1-set aw (Arquimed S.A, Santiago, Chile), was used to measure the *a_w_* of the different doughs and baked matrices at 25 °C. The device was calibrated using saturated salt solutions of known relative humidity, namely, 11.3%, 32.8%, 52.9%, 75.3%, and 90.1%. After calibration, 5 g of sample were placed inside the measuring chamber, and the head of the sensor was fitted to seal the chamber until equilibrium was reached and the reading of the detector was recorded. The *a_w_* values were obtained with ±0.01 accuracy [73].

#### 4.4.3. Textural Changes of Starchy Matrices before Baking

A texture profile analysis (TPA) of the dough was performed using a TA-XT plus texture analyzer with software texture expert exceed version 1.1.6 for Windows (Stable Micro Systems Ltd., Godalming, Surrey, UK). The device was equipped with a load cell capacity of 5 kg, a heavy-duty platform of aluminium (HDP/90) and a compression platen (P/100) made of stainless steel (100 mm diameter). Cylinder-shaped pieces of ~15 g of weight (10 mm of diameter and 15 mm of height) were obtained from the central part of the dough using a metal cutter [18,36,49]. These dough pieces were covered by a plastic film from the moment they were cut until they were analyzed to avoid dehydration. 

The tests were performed at 25 °C (room temperature) with a speed of 5 mm/s to compress the dough and up to 4.5 mm of their original height (30% strain level), using two-cycle uniaxial compression, simulating the human bite with a delay time of 10 s. The measurements were repeated six times for each dough [74]. In these experiments, a smaller deformation level was chosen, as, under a large deformation, the samples collapsed. The parameters used as indicators of textural changes in the dough were hardness (maximum resistance to the first compression peak, N), adhesiveness (negative force of the first compression cycle, N·s), cohesiveness (area under the second peak divided by area under the first peak), springiness (distance during the second compression separated by the distance during the first compression, as an approximation to measure the ability of the sample to recover its original form after the deforming force was removed), and chewiness (hardness × cohesiveness × elasticity, N). All variables were calculated from the TPA curve.

#### 4.4.4. Textural Changes of Starchy Matrices after Baking

The textural changes of the baked matrices were measured using a three-point bending test following the procedure described by Dueik and Bouchon [69], with some modifications. The analysis was carried out in a TA-XT plus texture analyzer (Stable Micro Systems Ltd., Godalming, UK), with a 5 kg cell. Each sample was placed on two parallel edges (considering a support span of 16 mm), in order to apply the load centrally. A 2.5 mm-thick steel blade with a flat edge was used to fracture the sample at a speed of 10 mm/s. Texture measurements of the samples were conducted at room temperature (25 °C). The maximum breaking force (*F_max_*) at the fracture point (the highest value in the plot) was obtained using texture expert software version 1.16 for Windows (Stable Micro Systems Ltd., Godalming, UK).

### 4.5. Chemical and Analytical Methods

#### 4.5.1. Determination of AA Content in Baked Starchy Matrices

The effect of SDF and GTP addition on AA formation in baked starchy matrices, was quantified by gas chromatography–mass spectrometry (GC–MS), using an Agilent 7890A gas chromatograph with a 5975 C mass-selective detector (Agilent Technologies Inc., Santa Clara, CA, USA), following the method developed by Mariotti-Celis et al. [21], Fu et al. [36], and Pacetti et al. [75], with some modifications.

All samples were milled before the extraction to fine particles using a mixer (model HR1617 650 W, Philips, China). Two grams of milled sample were transferred to a 50-mL centrifuge tube. Furthermore, 40 μL of d3-acrylamide were added as an internal standard to the centrifuge tube before 10 mL of methanol were added. Samples were placed in a vortex (model REAX top, Heidolph, Germany) during 30 s and left in an ultrasonic bath (model 970, VWR, Thorofare, NJ, USA), during 20 min at 60 °C to extract the AA content. Thereafter, the samples were centrifuged (model MIKRO 220R, Hettich, Germany) for 10 min at −4 °C and 6000 rpm (2817× *g*). Afterwards, an aliquot of 5 mL was cleaned through a C-18 reverse phase cartridge and a second extraction was made with 5 mL of methanol. Both extracts were combined and collected in a 100-mL balloon flask. Subsequently, the solvent was evaporated (Heidolph, model Hei-VAP advantage) until dryness and reconstituted with 1 mL of methanol. The eluate was transferred to Miniprep PTFE filter HPLC vials with a pore diameter of 0.20 m (Whatman Inc., Piscataway, NJ, USA), and put in 2-mL vials for GC–MS analysis.

AA content was determined by means of a linear calibration curve using standard solutions of AA dissolved in methanol (25, 150, 300, 450, 600, 750, 875, and 1000 pg/μL). Results were expressed as parts per billion (microgram of AA per kilogram of dry solids, i.e., μg AA/kg d.b.).

#### 4.5.2. Assessment of In Vitro Starch Digestibility in Baked Starchy Matrices

In vitro starch digestibility was determined in three stages, according to Englyst et al. [8] with some modifications. *Step 1*: Approximately 1.5 g of each sample were finely milled, using a food crusher (Oster^®^ OHB-126X, Milwaukee, WI, USA), and mixed in polypropylene tubes (50 mL) with 5 mL of a 50% saturated benzoic solution and 10 mL of pepsin-guar gum solution (5 g pepsin and 5 g guar gum in 100 mL HCl to 0.05 M). Guar gum was added to standardize the viscosity of the fluid. The pH was maintained between 2.3 and 2.6. Then, the tubes were vortex-mixed and kept in a water bath under gentle shaking, at 37 °C for 30 min to induce protein hydrolysis. Five mL of 0.5 M sodium acetate buffer solution (at 37 °C and about pH 5.2 ± 0.2) and five glass balls (~15 mm diameter) were added to each tube, which were then gently shaken and maintained in the water bath at 37 °C for 3 min. The pH was checked at the beginning of the intestinal digestion, and it fluctuated between 7.1 and 7.5. 

*Step 2*: Five mL of pancreatin-amyloglucosidase-invertase fresh enzyme mixture (18 g, 4 mL, and 60 µg, respectively, per 100 mL enzyme mixture) were added to each tube, which were capped with parafilm^®^, gently mixed and placed horizontally in the water bath at 37 °C, under shaking (137 rpm), in order to mimic peristaltic movements as indicated in similar in vitro digestion models [76,77]. Under these conditions, each tube was removed from the bath precisely 20 and 120 min after the enzyme mixture was added. The hydrolysis was stopped by vortex mixing 0.2 mL of the contents with 4 mL of absolute ethanol, which resulted in a fraction removed at 20 min (G_20_) and 120 min (G_120_), respectively. The tubes were vigorously vortex mixed for 1 min, placed in boiling water for 30 min, and cooled in an ice water bath for 15 min. 

*Step 3*: Ten mL of a potassium hydroxide solution (7 M) were added to the polypropylene tubes and placed horizontally in an ice water bath and shaken for 30 min. Afterward, 0.2 mL of the mix was added to 1 mL of acetic acid (1 M), and lastly, 40 µL of amyloglucosidase from *Aspergillus niger* solution (1:7 dilution) were added. The tubes were vigorously vortex mixed and kept at 70 °C in a water bath for 30 min, followed by 10 min in boiling water. The containers were allowed to cool down in an ice water bath for 15 min until they reached room temperature. Then 12 mL of absolute ethanol was added to obtain the total glucose (TG) fraction.

Glucose concentration in the different fractions was measured using a glucose oxidase and peroxidase assay kit GAGO-20 (Sigma-Aldrich, St Louis, MO, USA). The absorbance was quantified at 520 nm, using an ultraviolet (UV)–Visible spectrophotometer (model-2601, Beijing Instrument Industry Co., Ltd., Chaoyang, China). The three fractions RAG (rapidly available glucose), SAG (slowly available glucose) and UG (unavailable glucose) were calculated according to the following Equations (2)–(4):

(2) $$\mathrm{RAG}\;(mg/g\;total\;glucose)=\left(G_{20}\right)/\mathrm{TG}$$

(3) $$\mathrm{SAG}\;(mg/g\;total\;glucose)=\left(G_{120}-G_{20}\right)/\mathrm{TG}$$

(4) $$\mathrm{UG}\;(mg/g\;total\;glucose)=\left(\mathrm{TG}-G_{120}\right)/\mathrm{TG}$$

### 4.6. Statistical Analysis

The physicochemical analysis of the SDF, along with AA assays, as well as the in vitro starch digestibility were performed in triplicate. The TPA tests in the doughs and the breaking force measurements in baked starchy matrices were performed six times for each formulation. All results correspond to the arithmetic mean (±) standard deviation. One-way analysis of variance (ANOVA) was used to analyze significant differences in normally distributed datasets. While the non-normally distributed datasets were analyzed using the Kruskal–Wallis test. The differences between the mean values of samples were resolved using the honest significant difference (HSD) Tukey method at 95% confidence. The data were processed in the Statgraphics Centurion program version 16.2.04 (Stat-Point Technologies Inc., Warrentown, VA, USA).

## 5. Conclusions

Our results showed that it is possible to formulate new baked starchy matrices based on a gluten-starch mix, with the addition of soluble dietary fiber and green tea polyphenols. Through an adequate combination of the ingredients incorporated, along with a controlled baking process, it was possible to reduce the acrylamide content in starchy models by up to 64%. Furthermore, the addition of soluble dietary fiber led to a significant reduction in starch digestibility, lowering the levels of rapidly available glucose and increasing the unavailable glucose fraction, either with or without the inclusion of green tea polyphenols. These findings may be useful to develop healthier starchy baked food products with a low acrylamide content and a low glycaemic index according to new food intake trends.

## Figures and Tables

**Table 1:** Physicochemical properties of wheat starch and soluble dietary fibers used in dough formulation.

Components	Parameters *	
	Water-Holding Capacity (g Water/g Dry Solids)	Apparent Viscosity (mPa·s)
	3000 rpm (704× *g*) for 15 min	Solutions at 5% and 30 °C
Inulin (IN)	2.49 ± 0.23 ^b^	2.76 ± 0.47 ^a^
Polydextrose (PD)	1.96 ± 0.18 ^b^	5.49 ± 0.51 ^c^
Partially hydrolyzed guar gum (PHGG)	2.36 ± 0.19 ^b^	8.74 ± 0.68 ^d^
Wheat starch	1.85 ± 0.12 ^a^	3.46 ± 0.68 ^b^

* The different letters in each column denote significant statistical differences (*p* < 0.05). Data are means ± standard deviation (*n* = 3).

**Table 2:** Formulations used in the study.

Ingredients							
Product Code *	Wheat Gluten (g/100g Dry Solids)	Native Starch (g/100g Dry Solids)	SDF (g/100g Dry Solids of Starch)			GTP (g/100g Dry Solid of Starch)	Moisture (% Wet Basis)
			IN	PD	PHGG		
GS	12	88	-	-	-	-	40
GS-IN	12	80.5	7.5	-	-	-	38
GS-PD	12	80.5	-	7.5	-	-	38
GS-PHGG	12	80.5	-	-	7.5	-	38
GS-GTP	12	86.5	-	-	-	1.0	42
GS-IN-GTP	12	79.5	7.5	-	-	1.0	40
GS-PD-GTP	12	79.5	-	7.5	-	1.0	40
GS-PHGG-GTP	12	79.5	-	-	7.5	1.0	40

* GS: gluten-starch; SDF: soluble dietary fiber (IN: inulin; PD: polydextrose; PHGG: partially hydrolyzed guar gum); GTP: green tea polyphenols.
